# Statistical evaluation of worst-case robust optimization intensity-modulated proton therapy plans using an exhaustive sampling approach

**DOI:** 10.1186/s13014-019-1335-8

**Published:** 2019-07-19

**Authors:** Zhiyong Yang, Heng Li, Yupeng Li, Yuting Li, Yu Chang, Qin Li, Kunyu Yang, Gang Wu, Narayan Sahoo, Falk Poenisch, Michael Gillin, X. Ronald Zhu, Xiaodong Zhang

**Affiliations:** 1 Cancer Center, Union Hospital, Tongji Medical College, Huazhong University of Science and Technology, Wuhan, 430022 China; 2 Department of Radiation Physics, The University of Texas MD Anderson Cancer Center, 1515 Holcombe Blvd, Unit 1150, Houston, TX 77030 USA

**Keywords:** Intensity-modulated proton therapy, Robust optimization, Worst-case scenarios, Proton dose uncertainties

## Abstract

**Purpose:**

To assess the worst-case robust optimization IMPT plans with setup and range uncertainties and to test the hypothesis that the worst-case robust optimization strategies could cover most possible setup and range uncertainties in the real scenarios.

**Methods:**

We analyzed the nominal and worst-case robust optimization IMPT plans of seven patients with head and neck cancer patients. To take uncertainties into account for the dose calculation, we performed a comprehensive simulation in which the dose was recalculated 625 times per given plan using Gaussian systematic setup and proton range uncertainties. Subsequently, based on the simulation results, we calculated the target coverage in all perturbation scenarios, as well as the ratios of target coverage located within the threshold of eight worst-case scenarios. We set the criteria for the optimized plan to be the ratios of 1) the dose delivered to 95% (D95%) of clinical target volumes 1 and 2 (CTV1 and CTV2) above 95% of the prescribed dose, and 2) the D95% of clinical target volume 3 (CTV3) above 90% of the prescribed dose in worst-case situations.

**Results:**

The probability that the perturbed-dose indices of the CTVs in each scenario were within the worst-case scenario limits ranged from 89.51 to 91.22% for both the nominal and worst-case robust optimization IMPT plans. A quartile analysis showed that the selective robust optimization IMPT plans all had higher D95% values for CTV1, CTV2, and CTV3 than did the nominal IMPT plans.

**Conclusions:**

The worst-case strategy for robust optimization is adequately models and covers most of the setup and range uncertainties for the IMPT treatment of head and neck patients in our center.

**Electronic supplementary material:**

The online version of this article (10.1186/s13014-019-1335-8) contains supplementary material, which is available to authorized users.

## Introduction

In intensity-modulated proton therapy (IMPT), setup and range uncertainties can prevent scanning spots from matching properly to form a conformal dose distribution [1, 2]. To account for these uncertainties, robust IMPT plans can be constructed using worst-case robust optimization, which is now implemented in the Eclipse treatment planning system (Varian Medical Systems, Palo Alto, CA) [3]. The so-called worst-case strategy optimizes the minimum and maximum doses at each voxel with different setup and range uncertainties to keep the plan robustness [4–6]. This method is fast and convenient because only a limited number of perturbation scenarios need to be calculated. It has been used both as a strategy to implement uncertainties in the plan’s robustness-optimization process and as a tool to evaluate the robustness of the nominal plan [4, 7].

In the worst-case robust optimization, dose perturbations are usually performed by calculating the plan for eight worst-case perturbation scenarios (two scenarios for range uncertainties of ±3.5% and six for setup uncertainties of ±3 mm in the left-right (L-R), superior-inferior (S-I), and anterior-posterior (A-P) directions). In our center, these eight worst-case settings are gradually introduced into the clinical routine for the robust optimization [7, 8].

In some situations, the perturbation settings may be conflicted or insufficient. For example, the perturbation settings may comprise sometimes, for instance, the error on a perturbation direction, cannot be + 3 mm and − 3 mm at the same time; conversely, the perturbation settings may miss some scenarios, since setup uncertainties may happen in all directions simultaneously with range uncertainties [9, 10].

Several previous studies have used simulations to estimate and verify the robustness of proton plans. Casiraghi et al. simulated 14 and 85 perturbation scenarios for a nominal IMPT plan by using the dose-error-bar distribution algorithm and the Monte Carlo approach to evaluate the feasibility of applying the worst-case scenarios approach to estimate the plan robustness [7]. Lowe et al. also applied the dose-error-bar distribution algorithm to incorporate the effect of fractionation in the evaluation of IMPT plan robustness with regard to setup errors [11, 12]. Park et al. used the fast-proton-dose calculation algorithm and statistical analysis method to assess the effect of setup errors and range uncertainties on proton dose distributions and dose–volume histograms (DVHs) [13]. Van Der Voort et al. simulated the perturbation values for the scenarios to be included in robust optimizations and calculated the perturbation values to ensure adequate clinical target volume (CTV) coverage for fractionated IMPT treatments in the presence of prior known systematic and random setup errors and range error distributions [10]. However, it is still unknown whether the robust optimization settings of eight worst-case perturbation scenarios are adequate for the development of a robust IMPT plan covering most possible setup and range uncertainties.

Accordingly, in this study, we have two research objectives: First, we tested the plan robustness of the nominal and worst-case robust optimization IMPT plans by calculating all possible dose distributions of 625 perturbation scenarios; Second, we evaluated whether the robustness optimization settings of eight worst-case perturbation scenarios which represent the largest deviations of uncertainties are sufficient to cover all possible dose distributions by comparing the target coverage of 625 perturbation scenarios with that of eight worst-case scenarios. In order to simulate all possible perturbation scenarios, we use the Gaussian sampling function to simulate a large number of the systemic setup and range uncertainties. We calculated the perturbation dose of both the nominal IMPT plan and worst-case robust optimization plan using the fast proton dose calculation algorithm proposed by Park et al. [14], then compared the dose deviations obtained from the simulations between these two plans.

## Materials and methods

### Patient selection and treatment planning

We retrospectively evaluated the clinically approved, multi-field optimization IMPT plans of seven patients with head and neck (HN) cancers. Patient and target characteristics are shown in Additional file 1: Table S1. To control the impact of planning target volume (PTV) margin on the dose coverage, the treatment plans were re-optimized by a single planner using the CTV directly. The treatment plans were re-optimized with three beam angles (60°, 180°, and 300°) using the selective robust optimization algorithm, which was an altered worst-case robust optimization algorithm implemented in Eclipse version 13.7, as detailed in our previous research [3]. The prescription dose for all plans was 70 Gy relative biological effectiveness (RBE) to CTV1 (defined as gross disease plus a 1-cm margin) in 33 fractions; 63 Gy RBE to CTV2 (encompassing the high-risk nodal volume adjacent to gross disease in the neck) in 33 fractions; and 57 Gy RBE to CTV3 (encompassing an additional margin beyond CTV2 for patients with pharyngeal tumors and uninvolved nodes in the neck considered at risk of harboring subclinical disease) in 33 fractions [15]. The objective function of the CTV was computed for the worst-case situations, including setup uncertainties of ±3 mm and range uncertainties of ±3.5%. After re-optimization, the selective robust optimization IMPT plans were then evaluated for the worst-case situations to keep the D_95%_ of CTV1 and CTV2 above 95% of the prescribed dose, and the D_95%_ of CTV3 above 90% of the prescribed dose. The reduction of the dose requirement for CTV3 was made because of the difficulty of robust optimization in patients with bilateral CTVs [10] and the need to spare critical organs while sufficiently covering the targets. After the selective robust optimization, the same objective functions for CTVs and organs at risk (OARs) were used to optimize the nominal plan, excluding the range uncertainties of ±3.5% and setup uncertainties of ±3 mm. After optimization, all 14 plans were normalized to facilitate dose comparisons. The normalization point—CTV1 D98% of 70 Gy, where Dx% was defined as the lowest dose covering x% of the volume—was the same for both the nominal and the selective robust optimization plans. The D_98%_ of CTVs, D_1%_ of spinal cord and brain stem, and D_mean_ of the left and right parotids were recorded and reported.

### Simulation and calculation of perturbation scenarios

Our goal was to exhaustively investigate all possible dose distributions for the perturbation scenarios. We used the Gaussian distribution function to simulate the systematic setup and range uncertainties, which influenced the dose calculation for all fractions. All sampling uncertainties were assumed to be independent. The perturbation scenarios are created by sampling the setup and range uncertainties in all four freedoms simultaneously. The setup uncertainties were randomly drawn from the Gaussian distribution function, with the mean at the planning isocenter; the standard deviation was equal to 1.5 mm in the L-R, S-I, and A-P directions to simulate the systematic setup uncertainties. Range uncertainties were also drawn from the Gaussian distribution function, with the standard deviation equal to 1.75% of the nominal computed tomography (CT) number-to-stopping power ratio calibration curve. Hence, about 5% of the total perturbation scenarios were outside the setup uncertainties (from − 3 mm to 3 mm) and range uncertainties (from − 3.5 to 3.5%) of the worst-case scenarios. The sampling number for the Gaussian simulation perturbation scenarios was set at 625.

Even if the simulation counts are limited to 625 dose calculations per plan, calculation of doses with the treatment planning system is still time-consuming. Therefore, in this study, dose distributions were approximated using a fast dose calculation method [14]. This method approximated new proton doses from the pre-computed nominal dose distributions by taking into account the perturbation of the radiologic path length caused by an isocenter shift or range uncertainties. The perturbed dose was calculated for each scenario and DVHs were obtained. Dose-volume indices including the D_95%_ of the CTVs, D_1%_ of the brain stem and spinal cord, and D_mean_ of the left and right parotids, were also recorded.

### Statistical analysis

SPSS 24.0 software (IBM, Armonk, NY) was used for statistical analysis. *P* values of less than 0.05 were considered statistically significant. The DVHs of the nominal IMPT and selective robust optimization IMPT plans were compared using a two-sided Wilcoxon signed-rank test to examine the dose indices of targets and OARs.

### Comparison of target coverage for eight worst-case scenarios and 625 perturbation scenarios

We used the lowest D_95%_ value among the CTVs of the eight worst-case scenarios (including range uncertainties of ±3.5% and setup uncertainties of ±3 mm for 3 directions) for a certain nominal plan as a threshold to determine whether the target coverages for each of the perturbation scenarios were bound within the worst-case robust scenarios. The probabilities that the D95% values of the CTVs of the 625 perturbation scenarios were higher than the threshold were calculated to investigate whether the ratio of that the worst-case robust strategy could ensure adequate coverage of the target volume for all perturbation scenarios.

### Comparison of target coverage in the 625 perturbation scenarios and the 95% of the prescribed dose

We calculated the D_95%_ of CTV1, CTV2, and CTV3, as target coverage in our study. The ratio of the D_95%_ above 95% of the prescribed dose in all 625 perturbation scenarios was also calculated. To evaluate the plan robustness, we also calculated the medians and interquartile ranges of the D_95%_ of CTV1, CTV2, and CTV3 for 625 perturbation scenarios. The differences in target coverage between the nominal and selective robust optimization IMPT plans were also compared using a two-sided Wilcoxon signed-rank test.

## Results

A total of 4375 dose distributions from 7 patients were calculated and analyzed in this study. In order to present our results in a simple and clear way, we selected case #5, which had the largest CTV1 volume, as a representative case for demonstration.

Table 1 show that, in this case, the D_98%_ values for CTV1, CTV2, and CTV3 were not significantly different between the selective robust optimization IMPT plan and the nominal IMPT plan. However, the D_1%_ of spinal cord (42.86 Gy) and brain stem (45.36 Gy) in the selective robust optimization IMPT plans were significantly higher than that of the nominal IMPT plan (40.26 Gy, *P* = 0.028; and 43.82 Gy, *P* = 0.028).

**Table 1:** Summary of doses to targets and OARs

Parameter	Robust optimization IMPT Mean (SD)	Nominal IMPT Mean (SD)	*P* ^a^
CTV1, D_98%_ (Gy)	70.00 (0.00)	70.00 (0.00)	1.000
CTV2, D_98%_ (Gy)	64.66 (1.27)	64.46 (1.57)	0.735
CTV3, D_98%_ (Gy)	57.47 (0.72)	57.53 (1.05)	0.866
Spinal cord, D_1%_ (Gy)	42.86 (2.88)	40.26 (3.63)	0.028^*^
Brain stem, D_1%_ (Gy)	45.36 (8.82)	43.82 (9.41)	0.028^*^
Left parotid, D_mean_ (Gy)	37.13 (3.98)	36.00 (5.00)	0.398
Right parotid, D_mean_ (Gy)	37.46 (6.60)	36.72 (5.96)	0.310

*Abbreviations*: *CTV* clinical target volume, *D*_*x%*_ dose delivered to x% of the volume, *D*_*max*_ the maximum dose delivered to the structure, *D*_*mean*_ the average dose delivered to the structure, *OARs* organs at risk

^*^P values < 0.05 were considered statistically significant

^a^Comparison of the selective robust optimization IMPT plan and the nominal IMPT plan

### Comparison of target coverage among eight worst-case scenarios and 625 perturbation scenarios

Table 2 shows the probabilities that the dose indices of 625 perturbation scenarios were within the threshold of the eight worst-case scenarios in terms of target volumes/OARs. The comparisons of the thresholds of the eight worst-case scenarios show that the target coverages of CTV1 and CTV2 were significantly higher in the selective robust optimization IMPT plans (68.71 Gy, and 61.86 Gy, respectively) than in the nominal IMPT plans (67.51 Gy, *P* = 0.018; and 60.88 Gy, *P* = 0.018). For the 625 perturbation scenarios, the probabilities of the D_95%_ of CTV1 being above the threshold were 90.38% in the selective robust optimization IMPT plans and 91.22% in the nominal IMPT plans. The probabilities of the D_95%_ of CTV2 being above the threshold were 90.63% in the selective robust optimization IMPT plans and 89.51% in the nominal IMPT plans. The probabilities of D_95%_ of CTV3 being above the threshold were 90.63% in the selective robust optimization IMPT plans and 90.13% in the nominal IMPT plans. For OARs (spinal cord, brain stem, and left and right parotids), at least 94.86% of the 625 perturbation scenarios showed lower dose indices than those given by the maximum value of the eight worst-case scenarios.

**Table 2:** Statistical summary of the probabilities that the perturbed dose indices of 625 scenarios were within the worst-case scenarios

Parameter	The threshold of eight worst-case scenarios (Gy)			The probabilities of Gaussian simulation within the eight worst-case scenarios	
	Selective robust IMPT Mean (SD)	Nominal IMPT Mean (SD)	*P* ^a^	Selective robust IMPT Mean (SD)	Nominal IMPT Mean (SD)
CTV1, D_95%_	68.71 (0.49)	67.51 (0.85)	0.018^*^	90.38% (2.53%)	91.22% (2.05%)
CTV2, D_95%_	61.86 (1.49)	60.88 (1.44)	0.018^*^	90.63% (2.59%)	89.51% (4.47%)
CTV3, D_95%_	51.98 (3.27)	51.34 (3.18)	0.063	90.63% (3.78%)	90.13% (4.10%)
Spinal cord, D_1%_	48.07 (4.29)	47.19 (5.14)	0.398	96.01% (1.63%)	95.93% (1.96%)
Brain stem, D_1%_	50.84 (8.46)	49.92 (10.83)	0.398	97.01% (1.21%)	95.87% (1.54%)
Left parotid, D_mean_	40.22 (3.82)	39.14 (5.38)	0.398	95.68% (2.94%)	95.34% (2.50%)
Right parotid, D_mean_	40.63 (6.04)	40.61 (5.52)	1.000	94.86% (1.85%)	94.80% (2.00%)

*Abbreviations*: *CTV* clinical target volume, *D*_*max*_ the maximum dose delivered to the structure, *D*_*mean*_ the average dose delivered to the structure

^*^P values < 0.05 were considered statistically significant

^a^Comparison of the threshold of eight worst-case scenarios between the selective robust optimization IMPT plan and the nominal IMPT plan

**Fig. 1:**
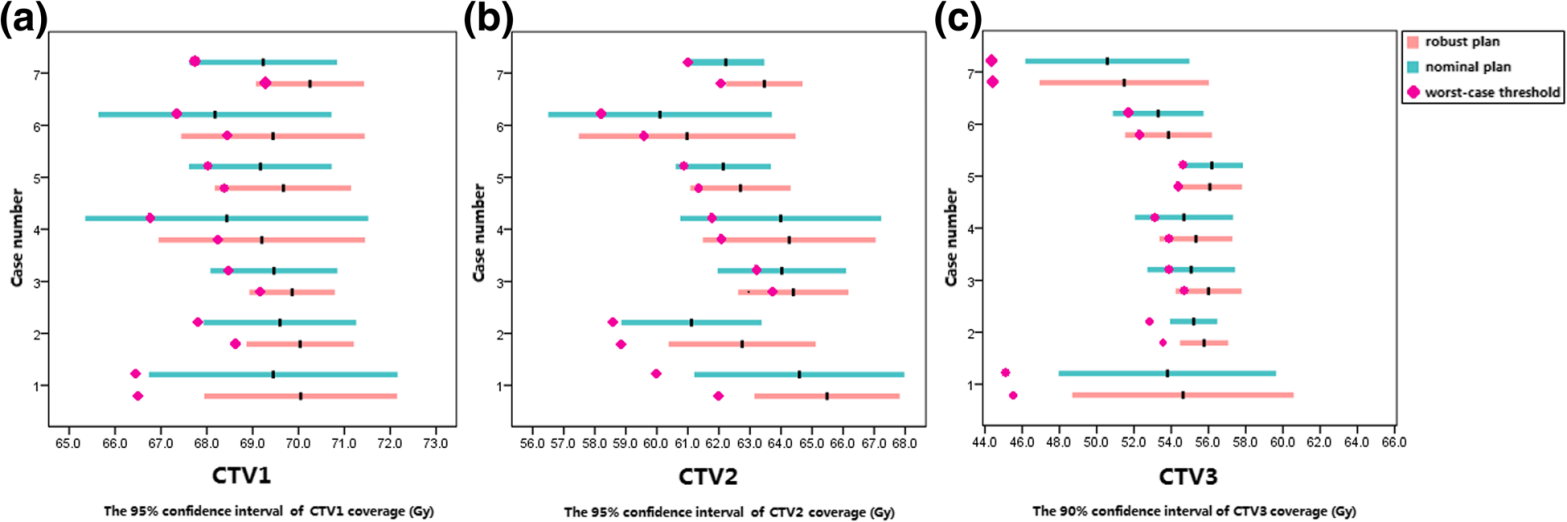
Boxplots for the selective robust and nominal plans show the confidence intervals of target coverage of 625 perturbation scenarios were shown for all seven cases. Sections **a** is CTV1, sections **b** is CTV2, and Sections **c** is CTV3

**Fig. 2:**
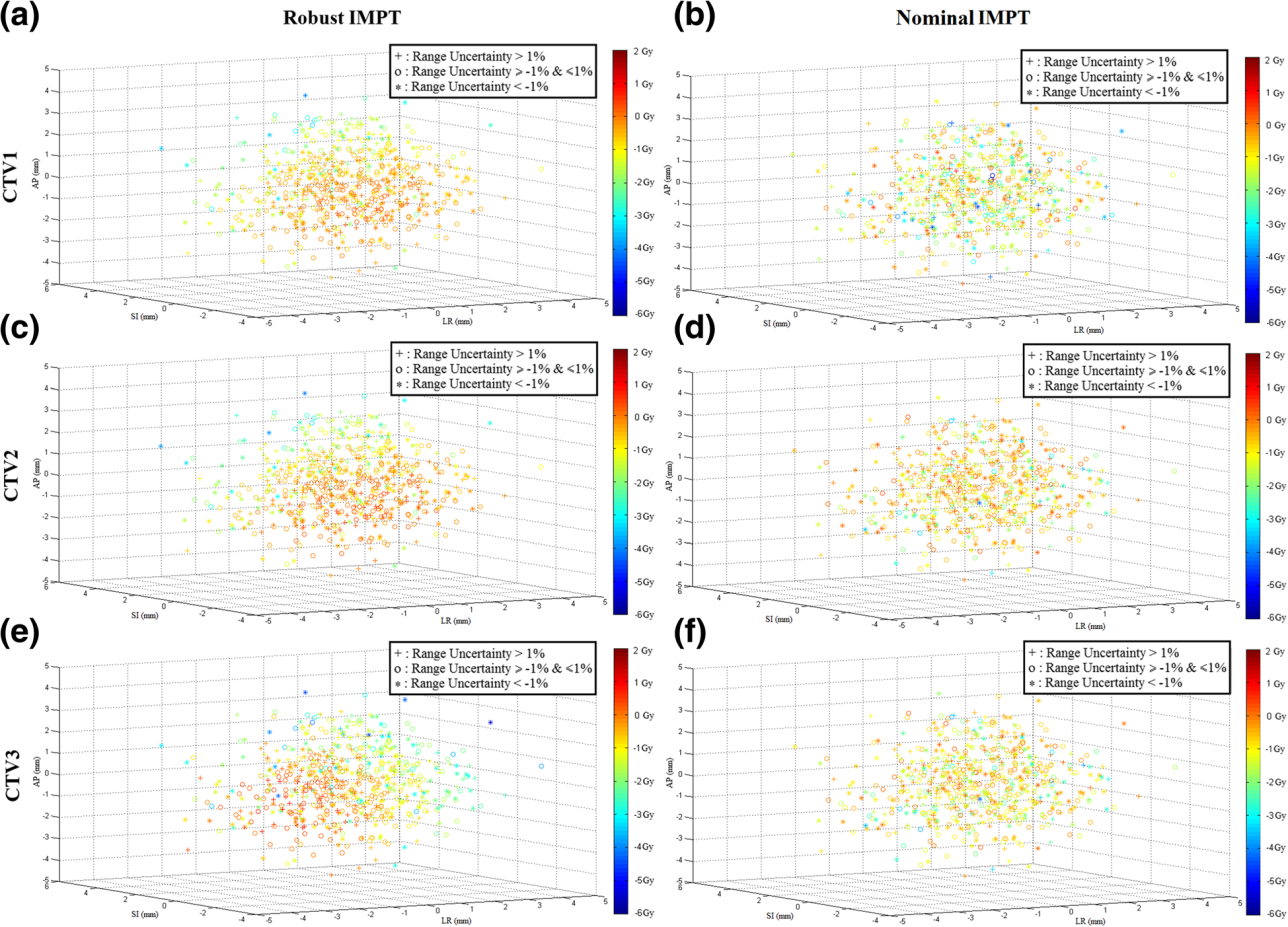
Dose deviation maps of targets for case 5. The dose deviations of CTV D_95%_ values between the nominal scenario and each perturbation scenarios are shown in the 3D setup error spaces [x direction, LR (left-right); y direction, SI (superior-inferior); z direction, AP (anterior-posterior)]. The dose deviations are scaled to the color bands (from -6Gy to 2Gy) and marked by different range uncertainty levels, located in the setup error coordinates. For all three CTVs of case 5, the selective robust optimization IMPT plan (**a, c, e**) shows fewer cold scenarios than the nominal plan (**b, d, f**)

The confidence intervals for clinical target volume coverage in the 625 perturbation scenarios and eight worst-case scenarios are shown in Fig. 1. For CTV1 and CTV2, the eight worst-case scenario thresholds for the selective robust optimization and the normal IMPT plans were similarly located within the 95% confidence interval range. For CTV3, the eight worst-case scenario thresholds for the selective robust optimization and the normal IMPT plans were similarly located within the 90% confidence interval range. These results suggest that the thresholds sufficiently evaluated the plan robustness for both the nominal and the robust optimization IMPT plans. The dose deviation maps of targets for case 5—the representative case—are shown in Fig. 2. The dose deviations were scaled to a color band and marked by different range uncertainty levels, located in the setup uncertainties coordinates. For all three CTVs for case 5, the selective robust optimization IMPT plan showed fewer cold scenarios than did the nominal plan.

### Comparison of target coverage of 625 perturbation scenarios and the 95% of the prescribed dose

**Fig. 3:**
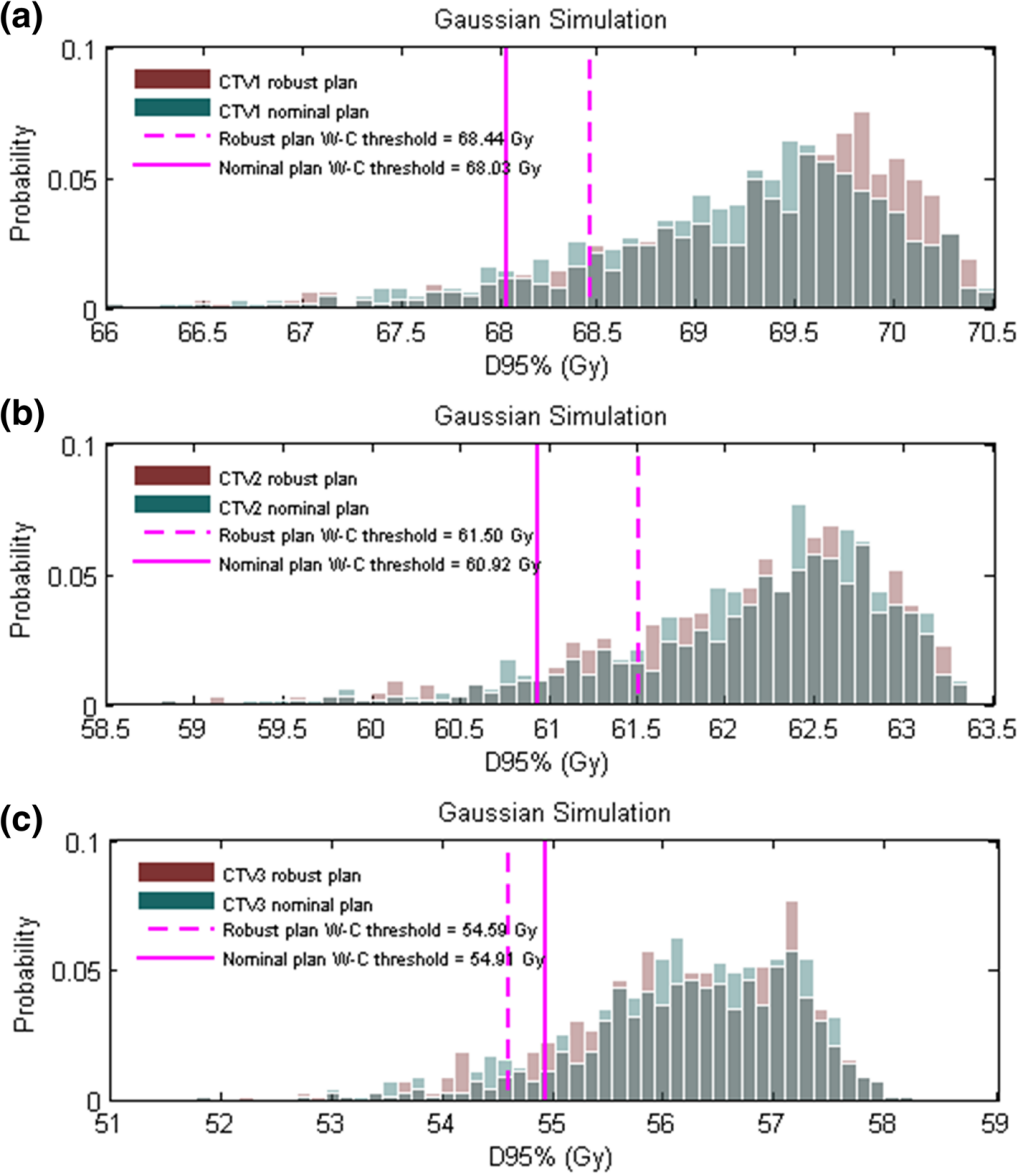
The probability distributions of D_95%_ values for Gaussian simulation strategies for CTV1 (**a**), CTV2 (**b**), and CTV3 (**c**) are shown for case #5. The probability distributions of the selective robust optimization IMPT plans (red bars) are seen in a higher dose area than that of the nominal plans (green bars). The thresholds of the eight worst-case scenarios for the selective robust optimization (pink dashed line) and nominal (pink solid line) IMPT plans are also shown

For all three CTVs evaluated, the ratios of the D95% of the CTV to 95% of the prescribed dose were significantly higher in the selective robust optimization plan than in the nominal plan (*P* = 0.043 for CTV1, 0.027 for CTV2, and 0.028 for CTV3), as shown in Table 3. The median values of D95% for CTV1, CTV2 and CTV3 of the selective robust optimization IMPT plans were 69.92 Gy, 63.60 Gy, and 55.10 Gy,. The median values of D95% for CTV1, CTV2 and CTV3 of the nominal IMPT plans were 69.24 Gy, 62.75 Gy, and 54.43 Gy. The median values of D95% for CTV1, CTV2 and CTV3 of the selective robust optimization IMPT plans were significantly higher than those of the nominal IMPT plans (*P* = 0.018, 0.018, and 0.028). For CTV1, the interquartile range of D95% of the selective robust optimization IMPT plans was smaller than that of the nominal IMPT plans (*P* = 0.018); there was no significant difference in interquartile ranges for CTV2 and CTV3. The probability distributions of the D95% values for all CTVs in case 5 are shown in Fig. 3, the CTVs D95% values of the selective robust optimization IMPT plans domain in higher dose area than that of the nominal plans.

**Table 3:** Ratio and quartile analysis of Gaussian simulations

	Robust IMPT Mean (SD)	Nominal IMPT Mean (SD)	*P* ^b^
CTV1 D_95%_			
Ratio^a^	96.96% (6.49%)	89.37% (21.25%)	0.043^*^
Median (Gy)	69.92 (0.35)	69.24 (0.53)	0.018^*^
Interquartile range (Gy)	1.02 (0.33)	1.34 (0.45)	0.018^*^
CTV2 D_95%_			
Ratio^a^	96.11% (8.57%)	91.29% (15.70%)	0.027^*^
Median (Gy)	63.60 (1.49)	62.75 (1.72)	0.018^*^
Interquartile range (Gy)	1.49 (0.50)	1.64 (0.61)	0.237
CTV3 D_95%_			
Ratio^a^	84.55% (14.64%)	72.94% (18.07%)	0.028^*^
Median (Gy)	55.10 (1.61)	54.43 (1.79)	0.028^*^
Interquartile range (Gy)	2.20 (1.23)	2.51 (1.31)	0.499

*Abbreviations*: *CTV* clinical target volume, *D*_*x*_ dose delivered to x% of the volume

^*^P values < 0.05 were considered statistically significant

^a^Ratio above 95% of the prescribed dose

^b^Comparison of the selective robust optimization IMPT plan and the nominal IMPT plan

## Discussion

In our center, we use the eight worst-case scenarios to evaluate the plan robustness of the HN IMPT plans with the eight worst-case scenarios before the treatment begins. The robustness evaluation criteria were D_95%_ of CTV1 and CTV2 above 95% of the prescribed dose, and D_95%_ of CTV3 above 90% of the prescribed dose. If the eight worst-case scenarios of the IMPT plan do not meet the plan robustness evaluation criteria, en attempt is made to re-optimize the IMPT plan with the robust optimization to improve the plan robustness. The objective of this study was to test the assumption that the eight worst-case scenarios can be used to evaluate IMPT plan robustness of HN cancers and to verify that the worst-case scenarios cover the possible real-world uncertainties. Our result showed that 89.51 to 91.22% of perturbed doses have CTV D95% values of higher than the threshold of eight worst-case scenarios for the setup and range uncertainties. In addition, for robust optimization IMPT plans, the probabilities of target coverage above 95% of the prescribed dose were 96.96% for CTV1, 96.11% for CTV2, and 84.55% for CTV3.

Our findings show that robust optimization and nominal IMPT plans retain about 90% of perturbation scenarios; in other words, the patient population to keep the CTV coverage (D95% of CTV) above the threshold of eight worst-case scenarios, as shown in Table 2. In a previous study, van Herk et al. [16, 17] considered a cumulative minimum CTV dose of 95% of the prescribed dose above 90% of the patient population to be an adequate for photon CTV-PTV margin. Our result shows a similar probability of the patient population for the CTV coverage of the threshold of eight worst-case scenarios. Unlike van Herk et al., who developed their simulations using ideal synthetic photon dose distributions that were not sensitive to real patient setup uncertainties, we calculated realistic proton dose distributions for both setup and range uncertainties using the fast-proton-dose calculation algorithm. Therefore, it was credible to apply the eight worst-case scenarios as a tool to evaluate the plan robustness of the nominal and robust optimization plans to the setup and range uncertainties in the IMPT treatment.

The robust optimization plans kept CTV1 and CTV2 coverage above 95% of the prescribed dose for 96.11 to 96.96% of the perturbation scenarios, as shown in Table 3. The target coverage for CTV1 and CTV2 also achieved the margin assumption of van Herk et al. [16, 17]. However, the coverage of CTV3 dropped approximately to 84.55%, across the patient group and in both the selective robust optimization and nominal IMPT plans. This reduction of CTV3 was expected, since the robustness evaluation setting for CTV3 was D_95%_ above 90% of the prescribed dose; in contrast, comparing to CTV1 and CTV2, dropped from 95 to 90% of the prescribed dose. This difference may be the result of the large anatomical changes of CTV3 in the lower neck, as mentioned in previous studies; for example, in a study by Van Der Voort et al., bilateral HN patients need a higher-range robust setting to cover the same degree of range uncertainties for their CTV-low volume (similar to CTV3 in our study) [10]. Sometimes, CTV1 and CTV2 are in the volume of the CTV3, but the CTV3 is adjunct to the OARs (such as spinal cord or parotids), and the dose gradient beyond CTV3 may be much higher than that beyond CTV1 and CTV2 [18]. This situation leads the target coverage of CTV3 to be less robust than that of CTV1 and CTV2 in our study could only keep 84.55% probability of target coverage above the threshold of the eight worst-case scenarios. This finding suggests that robustness settings need to be separately considered for various treatment sites due to the target shapes and anatomical changes.

In our center, nominal HN IMPT plans are still used in clinical practice, but we needed to confirm the plan robustness of the nominal HN IMPT plans using the eight worst-case scenarios before the treatment. In this study, we found that, if the nominal IMPT plans could satisfy the robust criteria (the D95% of CTV1 and CTV2 above 95% of the prescribed dose, and the D95% of CTV3 above 90% of the prescribed dose in the eight worst-case thresholds), 90% of all 625 scenarios of the target coverage of CTVs would be above the worst-case thresholds. These results suggest that the plan robustness of the nominal HN IMPT plans could still be retained above the eight worst-case thresholds for 90% of the patient population. This may be the result of the large spot size of the scanning system in our center (1σ, as measured in air at the isocenter: approximately 5 mm at the highest energy level and 10 mm at the lowest energy level when the energy absorber is used or approximately 14.5 mm without the use of the energy absorber) [19]. The large spot size may blur and reduce the sensitivity of the dose distribution to the uncertainties [20, 21].

One of the limitations of this study was that we did not apply robust optimization on OARs (e.g., the spinal cord and brain stem). In theory, the selective robust optimization methods can optimize the targets and OARs together under setup and range uncertainties. However, for HN cases, this may lead to some conflicts of the robust optimization objectives, since the OARs are usually adjacent to, or even overlapped with, the targets (e.g. parotids) [15]. Hence, our study was focused on the influence of worst-case robust optimization to targets and to set the selective robust optimization objectives only on targets. Second, we did not divide the setup uncertainties into system and random errors separately and only used the Gaussian sampling to simulate the systematic setup and range uncertainties directly. The systematic setup and range uncertainties influenced our dose calculation for all fractions. We did this because we are focusing on verifying the hypothesis that the dose distributions recalculated for the realization of the largest setup and range uncertainties could represent the largest deviations of the delivered dose from the nominal plan dose. Hence, we only simulated the systematic setup uncertainties that would be dominant and would impact the dose calculation of all fractions [13, 16, 17]. The random setup uncertainties might be obscured and averaged during the treatment fractions, they would have less influence on the target coverage than the systematic setup uncertainties [13]. Additionally, it is important to note that the results presented in this work may be affected by the dose calculation accuracy. Based on the previous study, the RMS of the difference in relative dose between the fast range-corrected proton dose approximate algorithm and the full 3D pencil beam convolution superposition algorithm of Eclipse TPS should be less than 1–2% [14, 22]. This accuracy of the DVH curve might still be clinically acceptable.

## Conclusion

In this study, we evaluated the assumption that the eight worst-case scenarios for robust optimization could retain the target coverage for systematic setup and range uncertainties. We found that the worst-case robust evaluation strategy is adequate to model the target coverage for possible setup and range uncertainties in both the nominal and robust optimization plans for HN patients in our center. Compared to the higher target volumes, CTV1 and CTV2, CTV in the neck, CTV3, may be less robust for IMPT and may loss some target coverage. This finding suggests that robustness settings need to be separately considered for the various treatment sites and that, our results may be appropriate only for HN patients. Further studies on various treatment sites are needed.

## Additional file

Additional file 1: **Table S1.** Patient and target characteristics. (DOCX 15 kb)

## Data Availability

Please contact author for data requests.

## Abbreviations

CTV1: Clinical target volume 1
CTV2: Clinical target volume 2
CTV3: Clinical target volume 3
DVH: Dose–volume histograms
Dx%: The lowest dose covering x% of the volume
HN: Head and neck
IMPT: Intensity-modulated proton therapy
OAR: Organ at risk
PTV: Planning target volume 2
