# Supplementary material for: Statistical evaluation of worst-case robust optimization intensity-modulated proton therapy plans using an exhaustive sampling approach
Source: Radiat Oncol. 2019 Jul 19;14:129. doi: 10.1186/s13014-019-1335-8 (PMC6642585; doi:10.1186/s13014-019-1335-8)
Supplement: Supplementary file 1 — Table S1. Patient and target characteristics. (DOCX 15 kb) [file 13014_2019_1335_MOESM1_ESM.docx]

Table S1. Patient and target characteristics

| Patient | Tumor location | CTV volume (cm3) | | |
| --- | --- | --- | --- | --- |
| number |  | CTV1 | CTV2 | CTV3 |
| 1 | Nasopharyx | 259.3 | 484.3 | 238.4 |
| 2 | Nasopharyx | 259.8 | 452.9 | 211.9 |
| 3 | Base of tongue | 231.8 | 450.0 | 378.0 |
| 4 | Base of tongue | 153.4 | 231.3 | 236.3 |
| 5 | Base of tongue | 270.9 | 484.5 | 205.9 |
| 6 | Base of tongue | 149.2 | 78.3 | 241.0 |
| 7 | Base of tongue | 173.5 | 387.2 | 252.6 |

Abbreviations: CTV = clinical target volume.
